# Performance Comparison of Geodrain Drainage and Gravel Drainage Layers Embedded in a Horizontal Plane

**DOI:** 10.3390/ma14216321

**Published:** 2021-10-22

**Authors:** Mariusz Cholewa, Karol Plesiński

**Affiliations:** Faculty of Environmental Engineering and Land Surveying, University of Agriculture in Kraków, Al. Mickiewicza 24/28, 30-059 Kraków, Poland; karol.plesinski@urk.edu.pl

**Keywords:** drainage geocomposite, gravel drainage, filtration speed, suction speed

## Abstract

Drainage materials are widely used, among other uses, in the construction of landfills. Regulations require a drainage layer in the base and a covering for the landfill. The implementation of a gravel drain requires a lot of material and financial outlays. New geocomposite materials are an alternative, and facilitate construction. The aim of the research was to compare the drainage properties of the Pozidrain 7S250D/NW8 geocomposite and gravel drainage. The model test was performed on a specially prepared test stand. The research was carried out for model #1, in which the gravel drainage was built. Model #2 had a drainage geocomposite built into it. The test results show the values of the volumetric flow rate for geodrains, with a maximum value of 40 dm^3^·min^−1^. For the gravel layer, values of up to 140 dm^3^·min^−1^ were recorded. Another parameter recorded during the damming of water by the embankment was the speed of water suction by the geosynthetic and gravel drainage; the values were 0.067 and 0.024 m^3^·s^−1^, respectively. The efficiency of water drainage through the geocomposite was sufficient. It is possible to use the slopes of the landfill for drainage, which will reduce material and financial outlays.

## 1. Introduction

Geosynthetics constitute a large group of chemical-based products manufactured mainly with (polymer) plastics, which have been widely used in ground construction works [1,2]. In engineering construction, it is mandatory for them to be listed in work specifications, rendering it possible to reduce material consumption and demand for transportation [3]. The beginnings of geosynthetics utilization were in the 1950s, conditioned by their physical, mechanical, and chemical properties [2,4]—and also, in some cases, by their small bulk weight [5]. Today, it is important that geosynthetics feature greater resistance to aging and biological processes compared to materials from previous years, owing to the employment of suitable additives in technological processes influencing the delay in their degradation. A wide range of synthetic materials’ applications influence the structure, solutions in ground constructions, and technology of their execution [6,7,8,9,10,11].

Physical and chemical structure condition geosynthetic properties; they may be modified at the production stage through admixing various additives, fillers, and softeners with the polymer, thus improving its usable properties. An advantage of a geosynthetic over a soil material is the combination of water permeability and tear resistance in one composite. Flexible plastic drainage pipes have been used in drainage systems for several decades; the pipe drains water through the holes made. It is necessary to prevent clogging with fine soil fractions. If the drained soil is very fine-grained, a geotextile cover is used. It is also possible to cover the soil with gravel. Clogging caused by biological, chemical, and mechanical stress is not a major problem.

**Figure 1:**
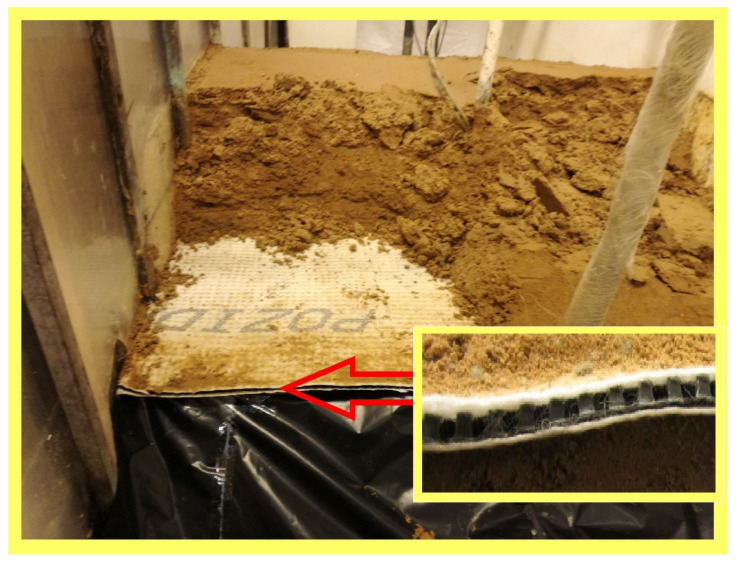
Drainage geocomposite with spatial structure in the form of an embossed core.

Since the hydrotechnical construction sector requires the employment of various types of filtration materials, which is connected with high demand for means of transportation, a search for modern solutions is underway [12]. Particular interest was spurred in the so-called drainage geocomposites and their spatial structure (Figure 1).

The aim of our research and analysis was to determine the drainage properties of a geocomposite with a cellular structure. The model tests carried out under laboratory conditions verified the water flow characteristics. The results of the maximum flow rate were obtained at the assumed water level. The results were compared with the values for the gravel layer. Research on geocomposites was carried out in order to confirm a given functionality. In earthwork, they perform very different functions. The applicable test standards may omit certain characteristics due to the excessively complicated testing method. The conducted research contributes information about the velocity in the suction zone to the geocomposite and gravel drainage, and presents the results of the volumetric flow rate through the tested materials.

As discussed in the literature, the use of geosynthetics in ground construction has become more profitable, both in technological and economic terms [13]. In Europe, in 2014, the consumption of geosynthetics amounted to 180 M m^2^ of geofabrics, 75 M m^2^ of geotextiles, 35 M m^2^ of geonets, 15 M m^2^ of geocomposites (including geodrains), 45 M m^2^ of geomembranes, and 20 M m^2^ of geosynthetic silt barriers. 

Values of hydraulic parameters of geotextiles are conditioned by many factors, e.g.,:

- The structure of a geotextile;
- The type of soil used with geotextiles in a specific structure;
- The hydraulic gradient;
- The mechanical, chemical, and biological warping of land [3].

The basic factor influencing the structure of a geosynthetic is the manufacturing technology (cut fibres, continuous fibres, needling, weaving, knitting, etc.), but at the same time, it exerts little influence on the anisotropy of hydraulic parameters, due to the probability distribution of geofabric pores’ occurrence.

When defining hydraulic and mechanical parameters, a tremendous role is played by the shape of grains (sharp-edged or cobble-shaped) and grain-size analysis of the soil used with a geosynthetic. A very important factor is the influence of soil on the characteristic size of geosynthetics’ pores, the friction coefficient between a polymer and mineral soil, and damage generation. Such parameters may be defined during laboratory research [14,15]. 

An important factor influencing water flow in geotextiles and geocomposites is the hydraulic gradient. The definition of water permeability in soil mechanics is based on Darcy’s law [16]. Pursuant to European standards, in the case of geosynthetics, the traditional notion of water permeability being connected with the hydraulic gradient equalling zero has been abandoned. There have been two assumptions made: of streamline and turbulent flow, as well as of a flow gradient that does not necessarily equal zero.

In the event of a mechanical warping of land, the most important factor is the grain size composition of the soil neighbouring a geosynthetic. It is possible that an arching emerges at the contact point with the material, or that finer soil particles penetrate the structure of a geosynthetic, which would limit water flow. On the other hand, chemical and biological warping of land may be spurred by a high salt content and the presence of microflora in the water.

## 2. Materials and Methods

For the research purpose, Pozidrain 7S250D/NW8 geocomposite drainage was used (ABG Ltd., Meltham Mills Road, Meltham, UK). Geodrains are usually made of a geofabric layer with a spatial structure, owing to which it is possible to intake large volumes of water and drain it rapidly [17,18]. The external sheathing—at least from one side—is made of a water-permeable geofabric that screens fine soil particles and prevents them from passing through the internal structure of the geodrain. Two external layers are permanently interconnected with adhesive bonding or thermal welding methods, making the product’s structure stable [5,19].

Drainage geocomposites are expected to be, to a certain degree, resistant to the influence of aggressive factors, as well as to feature high mechanical resistance. Their benefit is in their ease of assembly. Ground preparation works are limited to levelling, compacting, and removing elements that might pose a risk to the geosynthetic, and overlapping sheets of material do not require subsequent fixed bonding. An element that is constant and inherent to the structure of geodrains is the internal spatial structure, but there is also the possibility of using geocomposites, featuring one side made of an impermeable geosynthetic.

In the research, a drainage geocomposite was used, featuring 8.5 mm thickness and composed of three layers:

- Needled geofabric made of cut fibres with 1.2 mm thickness;
- A drainage core embossed in an HDPE sheet;
- A second needled geofabric with 1.2 mm thickness.

The materials were permanently joined with adhesive bonding and thermal welding. The geocomposite’s characteristics are presented in Table 1.

Model research of flow rate was performed on a specially prepared test station, with internal dimensions of 600 cm (length), 100 cm (width), and 120 cm (height). Water supply control and filtration expenditure measurement were rendered possible by means of a system of tubes and overflows [20,21].

**Figure 2:**
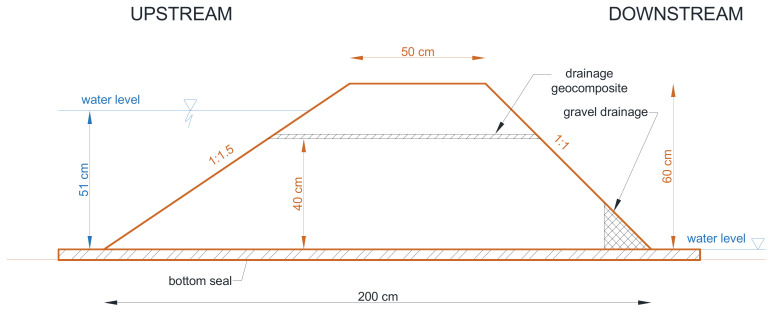
Cross-section of the model embankment with embedded geosynthetic.

**Figure 3:**
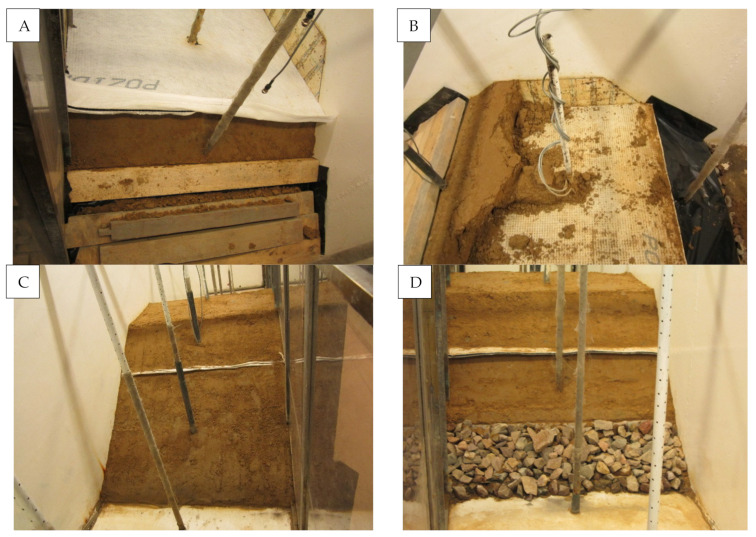
View of the model embankment with embedded geosynthetic; (**A**,**B**) embankment composition: (**A**) laying of geosynthetic and slope profiling; (**B**) covering of geosynthetic with a layer of soil, forming an embankment crown, protecting slopes against scattering; (**C**,**D**) after embankment execution: (**C**) waterside slope; (**D**) landside slope.

A 100 × 110 cm geodrain sample was embedded in a mineral soil featuring the parameters set out in Table 2. It was established that the embankment soil filtration coefficient is small, and does not influence the obtained filtration expenditure values for geodrain. This assumption was based on previous research conducted in the Department of Hydraulic Engineering and Geotechnics of the University of Agricultural in Kraków [22]. The mineral soil was formed as an embankment mechanically compacted to I_S_ = 0.95. Under the geodrain, the thickness of the soil amounted to 0.4 m, and to 0.2 m over the geodrain. Figure 2 presents the geometrical dimensions of the model cross-section. The model width was 1.0 m. The model was laid on a silt foundation with a thickness of 4 cm, replacing an impermeable foundation; its task was also to eliminate ground filtration. The model was protected against wall filtration by vertical silt strips. Figure 3 shows an overview of the embankment’s slope directly after its formation and before experimental tests.

**Figure 4:**
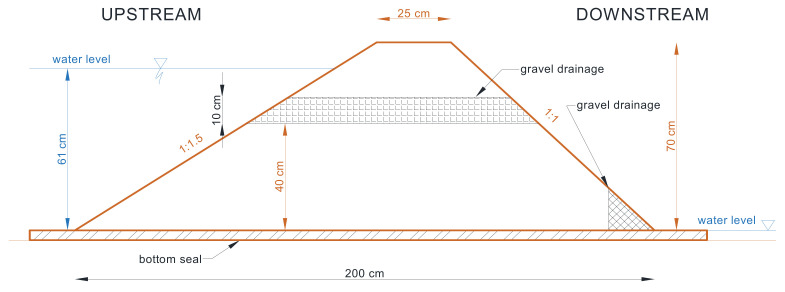
Cross-section of the model embankment with embedded gravel drainage covered with a geotextile.

**Figure 5:**
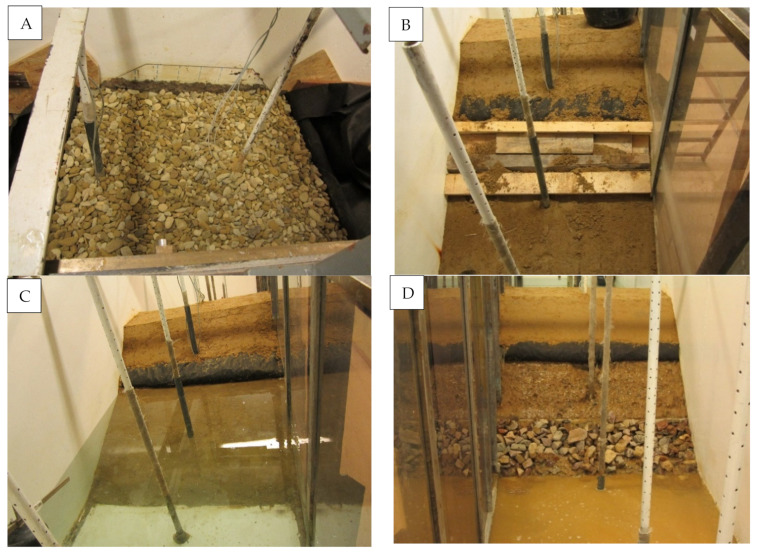
View of the model embankment slopes with embedded gravel drainage covered with a geotextile; (**A**,**B**) embankment composition: (**A**) gravel drainage forming; (**B**) profiling and securing slope banks; (**C**,**D**) after embankment execution: (**C**) waterside slope; (**D**) landside slope.

In model #2, the drainage geosynthetic layer was replaced with a horizontal layer of gravel drainage (Figure 4). The layer thickness amounted to 10 cm, and gravel was separated from the embankment soil with needled geofabric. The gravel was composed of cobble-shaped grains of 32–64 mm. The water permeability of the gravel exceeded 0.0025 m·s^−1^ (Figure 5).

The weight of the separating geofabric was 100 g·m^−2^, water permeability perpendicular to the geofabric plane was 125 mm·s^−1^, and assumed pore size (O_90_) was 120 μm.

Hydrodynamic measurements were taken with the use of the VALEPORT Model 802 Flat EM Flow Meter (Totnes, Devon, United Kingdom) hydrometric current meter, which allows for transient flow velocity measurements at a freely selected point in a cross-section. The velocity measurement range was V = 0.001 m∙s^–1^–10 m∙s^–1^, accurate to 0.001 m∙s^–1^. 

**Figure 6:**
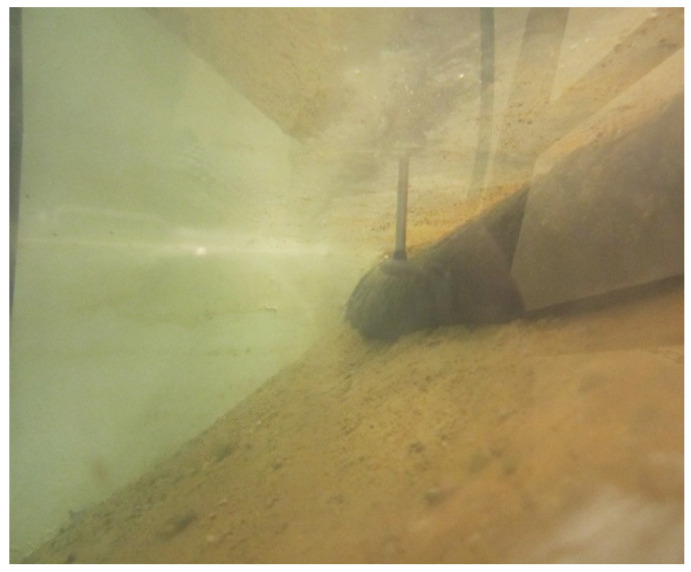
Sample flow velocity measurement at a location where water is sucked in by gravel drainage. The inlet to the drainage is protected by a geotextile against the suction of fine soil particles leading to clogging of the drainage geosynthetic.

Transient velocity measurements were taken at the location where water was sucked in by the drainage composite (Figure 6) and gravel drainage, at the waterside slope, at three points of the cross-section, located at ¼, ½, and ¾ of bed width from the left-hand side (denoted on the charts as L—embankment left-hand side, C—centre, and R—right-hand side, respectively). 

Each measurement was taken within a distance of 1 cm from the research object. Measurements were taken for 300 s at each point. Subsequently, the following calculations were made:

- Mean velocity at a measurement point [m·s^−1^]: $V_{ax,x}=\frac{V_{1}+V_{2}+V_{3}+\ldots +V_{300}}{V_{300}}$
- Mean velocity in a cross-section [m·s^−1^]: $V_{av}=\frac{\sum V_{av,x}}{3}$
- Flow rate [m^3^·s^−1^]: $Q=V_{av}\cdot A$

where:

x = L, C, R (measurement at the embankment’s left-hand side, in the centre, and at the right-hand side, respectively).
$V_{1},\ldots ,V_{300}$ —transient velocity measurement in the 1st, …, 300th second.

## 3. Results

**Figure 7:**
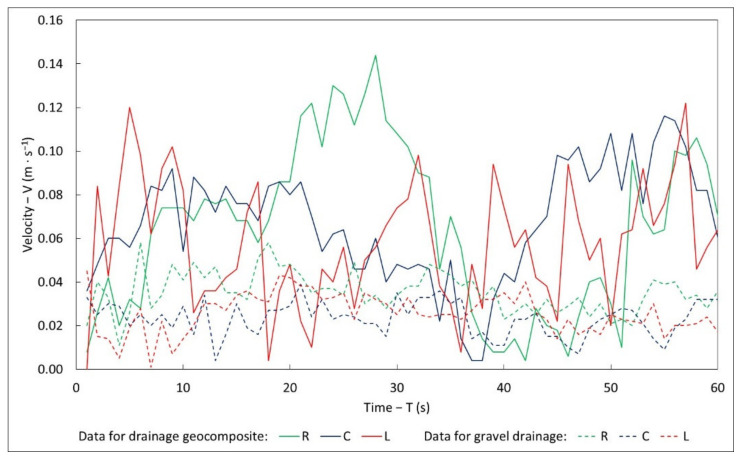
Section of a 60 s transient velocity measurement result from a 0.18 m column of water over the top plane of the research objects (for drainage geocomposite and gravel drainage).

Figure 7 presents a 60 s section of transient velocities measured in the zone of water suction, with a filling of 0.18 m over the top plane of the research objects.

**Figure 8:**
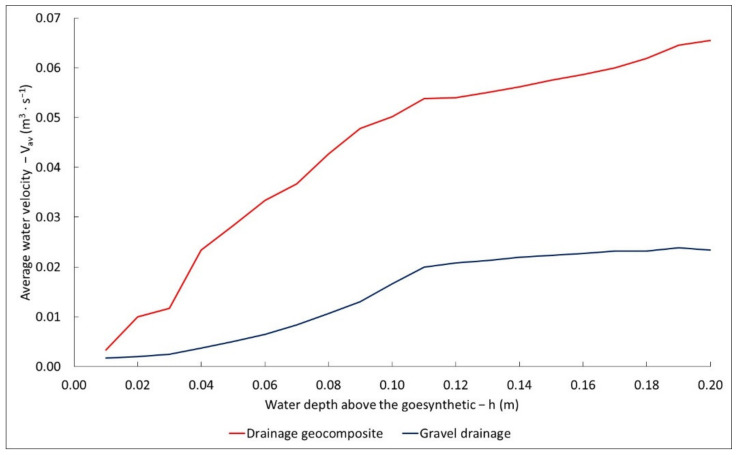
Water suction velocity of the geosynthetic drainage and gravel drainage for the entire cross-section.

Figure 8 shows averaged water suction velocities for geosynthetic and gravel drainage. It may be noted that the water flow rate through the geocomposite was much higher than through the gravel drainage. This is conditioned by a very small active surface of the geocomposite, through which water may flow; in this case, it amounted to 0.01 m^2^. In the case of gravel drainage, the active area amounted to 0.1 m^2^.

**Figure 9:**
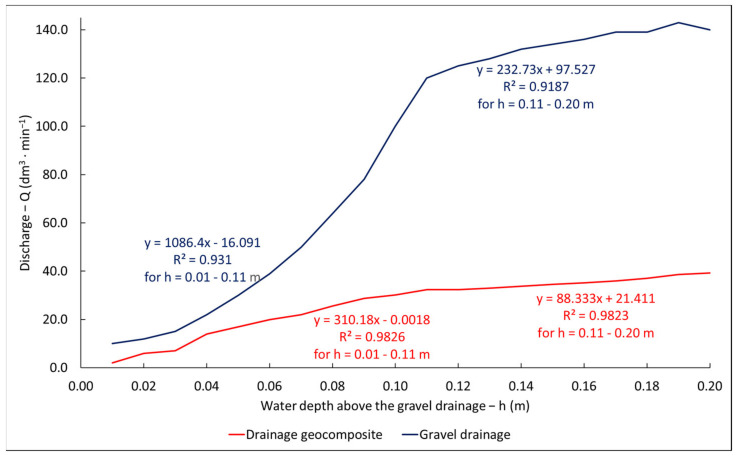
Influence of damming height on flow volume in the research objects.

Figure 9 shows the increase in flow rate when the water level increases. The water was dammed to 0.2 m over the geosynthetic plane. The graph presents averaged values from a number of measurements for a given water level. In the range of levels of 0–0.11 m over the geosynthetic plane, a rapid increase (of 2.8 dm^3^∙min^−1^) in flow up to 33 dm^3^∙min^−1^ was noted. With subsequent damming, flow volume increases are much slower (0.8 dm^3^∙min^−1^). For the water level of 0.2 m over the geosynthetic plane, a flow close to 40 dm^3^∙min^−1^ was noted. Further water level increases resulted in water overflowing over the model embankment crown. In the case of the drainage, as with the case of the geosynthetic, up to the height of 0.11 m of the column of water over the top plane of the object, a rapid flow increase (of 10 dm^3^∙min^−1^) up to 120 dm^3^∙min^−1^ was noted. During subsequent damming, the flow rate increase was slower, amounting to 2.2 dm^3^∙min^−1^. At the maximum water-damming level, the water flow rate through the gravel drainage amounted to 140 dm^3^∙min^−1^.

The mineral soil used for the construction of the embankment had a very good particle size distribution, U = 16.96. The soil could be easily compacted to I_S_ = 0.95. The total content of the clay–dust fraction was 21.38%. The above features made it possible to build a water-impermeable embankment. The geocomposite or gravel drainage layer was the only filtration route for the water. It can be assumed that the inflow was only in the horizontal plane, including after the water level was raised above the arranged drainages. If more permeable soil was used for the construction of the embankment, k_10_ > 1 × 10^−6^ m·s^−1^, the results would change. If we raised the water level above the gravel drainage, a significant amount of water would flow in from the bottom and top (water filtration through the soil). The geocomposite has an impermeable bottom layer; therefore, the inflow will be only from the ground zone above. The volume flow rate and the suction speed of the water are likely to be higher. This requires further experiments. The use of water-permeable soil will enable more detailed studies of the behaviour of drainage materials.

## 4. Summary

Geodrains play an important role in building structures, effectively and rapidly draining rainfall, infiltration, and inflow waters. Assignment of the aforementioned functions for drainage geocomposites must be backed by suitable research. Excessive or prolonged impact of water usually leads to the emergence of unfavourable phenomena; thus, there is a need to draw attention to the methodology of research on liquid flow in the plane of commercially available geocomposites. In technical datasheets, parameter values are obtained under the pressure in contact with hard–hard objects—thus, for example, when a geocomposite is found between two steel plates. This method deviates from the actual performance of a mineral soil, which is a plastic material. Only tests with soft–soft objects, in which standardised sponges are used [25], give valid results of liquid flow rates in a geocomposite under soil pressure. Execution of tests in this manner is consistent with the standard described in [26]. The results that are the closest to real-life conditions are obtained in tests in which a geosynthetic is found in direct contact with a soil. On a semi-technical scale, such model testing may provide evidence for the right research ideas. Laboratory studies of drainage geocomposites are presented in [27]; the authors examined the properties of a geocomposite consisting of a geotextile, geomembrane, and perforated tubes with a diameter of 10 or 20 mm. They obtained results confirming the good properties of drainage geocomposites. Practical and economic factors suggest their use in the drainage layers of landfills.

The drainage geocomposite used in our research has a cell structure. The cells form channels that carry water. The channels are not separated from one another. Water can move freely throughout the geocomposite. The gravel drainage layer has similar properties. The spaces between the gravel grains allow the water to move from zones of higher pressure to areas where flow is easier.

Drainage materials are widely used in the construction of landfills. The regulations require a base layer and a landfill cover. The implementation of the gravel drain requires a lot of material and financial outlays. New geocomposite materials are an alternative to facilitate construction, and their water drainage is sufficient. Placing a landfill on the slopes is of great help, and saves money.

## Figures and Tables

**Table 1:** General technical characteristics of the drainage geocomposite Pozidrain 7S250D/NW8.

Parameter	Unit	Value
Physical and mechanical features		
Thickness at 2 kPa pressure	mm	8.5
Mass per unit area	g·m^−2^	1040
Tensile strength	kN·m^−1^	24/19
Hydraulic properties		
Vertical water flow at 50 mm column of water	l·m^−2·^s^−1^	103
Water flow in the product’s plane at 20 kPa pressure HG = 1.0 *	l·(m·s) ^−1^	2.40
Water flow in the product’s plane at 100 kPa pressure HG = 1.0 *	l (m·s) ^−1^	1.95
Water flow in the product’s plane at 200 kPa pressure HG = 1.0 *	l (m·s) ^−1^	1.45

* Test with soft sponges simulating geotextile being embossed into a core by the soil.

**Table 2:** Basic geotechnical parameters of the soil material.

Parameter	Unit	Value
Fraction content according to [23] ▪ Gravel—2–63 mm ▪ Sand—0.063–2 mm Dust—0.002–0.063 mm ▪ Silt—≤0.002 mm	(%)	17.74 60.88 18.75 2.63
Soil type according to [24] standard	(–)	grsiMSa
Heterogranulation index U	(–)	16.96
Natural humidity (w)	(%)	7.95
Bulk density (ρ)	(g·cm^–3^)	2.16
Filtration coefficient k_10_ (for I_S_ = 0.95)	(m·s^–1^)	5.18 × 10^−8^

## Data Availability

Data sharing is not applicable to this article.
